# Supplementary material for: Optimal marker gene selection for cell type discrimination in single cell analyses
Source: Nat Commun. 2021 Feb 19;12:1186. doi: 10.1038/s41467-021-21453-4 (PMC7895823; doi:10.1038/s41467-021-21453-4)
Supplement: Supplementary file 1 — Supplementary Information [file 41467_2021_21453_MOESM1_ESM.pdf]

Supplementary information for scGeneFit : Label-guided optimal selection of target genes in scRNA-seq

Supplementary Note 1. Software package scGeneFit

We produced a Python package that implements the methods introduced in this paper. The package can be installed as

```
pip install scGeneFit
```

and it is available for Python 3.7 or later.

Our main method `get_markers` requires the following set of parameters:

- `data`: $N \times d$ numpy array with cells genetic expression, N: number of cells (i.e. "points"), d: number of genes (i.e. "dimension").
- `labels`: list with labels (N labels, one per point)
- `num_markers`: target number of markers to select (`num_markers`$< d$).
- `method`: 'centers', 'pairwise', or 'pairwise_centers'
  - 'centers' considers constraints that require that two consecutive classes have their empirical centers separated after projection to the selected markers. According to our numerical experiments this is the least general but most efficient and stable set of constraints (the underlying assumption is that the classes are linearly separable, which seems to be true in high dimensions).
  - 'pairwise' considers constraints that require that points from different classes are separated by a minimal distance after projection to the selected markers. Since all pairwise constraints would typically make the problem computationally too expensive, the constraints are sampled (sampling_rate) and capped (n_neighbors, max_constraints).
  - 'pairwise_centers' after projection to the selected markers every point is required to lie closest to its empirical center than every other class center (sampling and capping also apply here).
- `epsilon`: constraints will be of the form expr $> \Delta$, where Δ is chosen to be epsilon times the norm of the smallest constraint (default 1). This is the most important parameter in this problem, it determines the scale of the constraints, the rest the rest of the parameters only determine the size of the LP to adapt to limited computational resources. **We include a function that finds the optimal value of epsilon given a classifier and a training/test set. We provide an example of the optimization in** `https://github.com/solevillar/scGeneFit-python/blob/master/examples/scGeneFit_functional_groups.ipynb`
- `sampling_rate`: (if method=='pairwise' or 'pairwise_centers') selects constraints from a random sample of proportion sampling_rate (default 1)

- `n_neighbors`: (if method==‘pairwise’) chooses the constraints from n_neighbors nearest neighbors (default 3)
- `max_constraints`: maximum number of constraints to consider (default 1000)
- `redundancy`: (if method==‘centers’) in this case not all pairwise constraints are considered but just between centers of consecutive labels plus a random fraction of constraints given by redundancy. If redundancy==1 all constraints between pairs of centers are considered
- `verbose`: whether it prints information like size of the LP or elapsed time (default True)

We implemented a search in the parameter space using dual annealing to find the a good set of parameters for scGeneFit.

Supplementary Note 2. Numerical performance of scGeneFit

In this section we consider an expanded set of simulations in which we compare and contrast `scGeneFit` and one-vs-all marker selection procedures.

One dataset, multiple possible clusterings: heteroskedastic normal case

We consider the case where the same dataset might allow for multiple clustering, each with it respective sets of markers. To this end consider a small synthetic example with $n = 1000$ cells and $d = 200$ genes. We considered three different labelings of these n cells and assigned 50 genes as markers of each partition and another 50 genes as non-markers. Within each of the three labelings, the 50 markers for each label are drawn from a multivariate Gaussian with a 50 dimensional mean and a dense 50×50 covariance matrix. The 50 genes that are not markers are drawn from a univariate Gaussian distribution with mean 0 and variance 1. We set the marker set size to 20 and include as input one of the three labelings. We find that scGeneFit recovers markers of the appropriate labeling. Notably, since the third labeling is a finer partition of the first labeling, our method selects markers for both the first and third labelings when given the first labeling, but not vice versa. Discriminative markers were correctly recovered by scGeneFit for simulated samples drawn from mixtures of Gaussians corresponding to three label sets with two (Supplementary Figure 1 A), three (Supplementary Figure 1 B), and four (Supplementary Figure 1 C) labels, respectively. The probability of observing k markers for a given clustering is bounded above by $(50/1000)^k =$, k itself has too be large enough to allow for covariance estimation, and it is thus expensive.

Synthetic functional group model

We consider a synthetic model based on the intuition that genes interact with one another in a complex manner (e.g., their products belong to related pathways) giving raise to biological modules or functional groups which are utilized differently across cell types. Here, we consider functional groups to be determined by a (possibly overlapping) set of genes. Every functional group has a multi-modal structure: groups of genes can have different levels of activity (subgroups). Cell types are then determined by a unique combination of the different instances of the functional groups (see Supplementary Table 1 and Supplementary Figures 2, 3 for a specific example). To allow for clear visualization and metric evaluation, we consider a small toy example with 9 cell types and only 100 cells. The small toy example makes it easier to observe the effect the marker selection at the individual cluster level. In particular, we compare and contrast the performance of the two approaches in terms of true positive and false

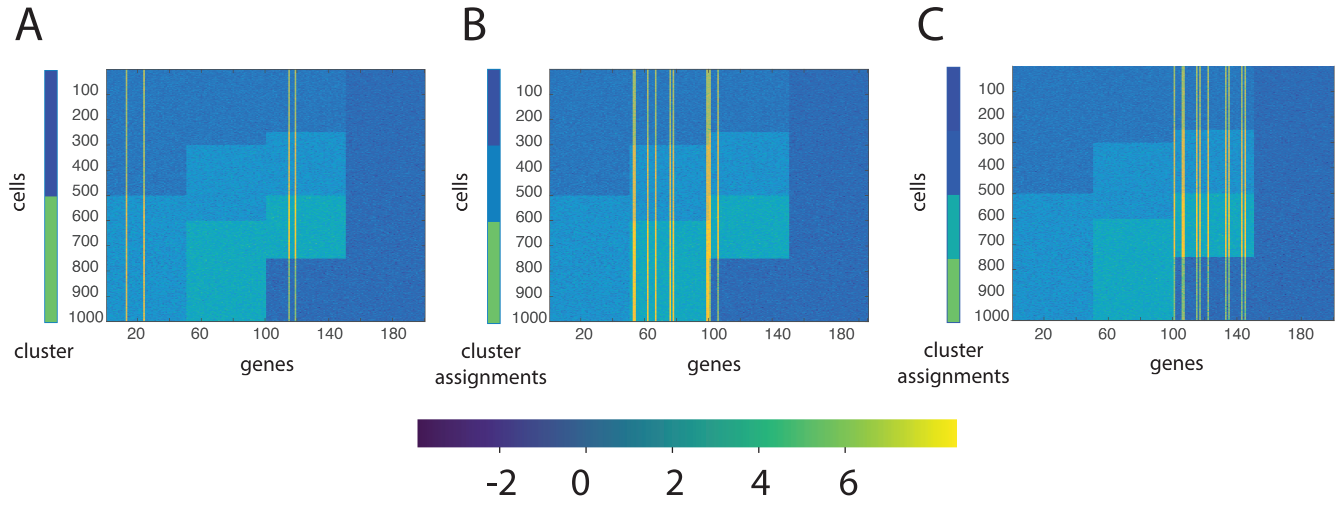

Supplementary Figure 1: Discriminative markers were correctly recovered by scGeneFit for simulated samples drawn from mixtures of Gaussians corresponding to three label sets with two (A), three (B), and four (C) labels, respectively. Each row is a single sample and each column is a single feature, and the shade of blue corresponds to the label assignment, with samples with the same label sharing a color. The yellow lines correspond to the markers selected by scGeneFit

positive rates, as illustrated by Receiver-operating characteristic curves. This shows that while scGeneFit optimizes for joint separation, it is able to outperform one-vs-all for most synthetic cell types in one-vs-all tasks (which, by design, should be favorable to the one-vs-all marker selection procedures).

Single cell count data

In Supplementary Figure 4, as suggested by the reviewers, we consider a count based model. The data is generated using a Poisson link function $\log(\texttt{Poisson}(\exp(X_{ij}))) + 1$, element wise, where X_{ij} is an element of the matrix X, generated from the mixture of multivariate normal distributions defined by the functional group example (Supplementary Figures 2 and 3). This follows statistical models with a rich history in describing count data [1, 2]. In Supplementary Figure 5, a similar model is explored in a real dataset scale setting.

Large scale computation

In Supplementary Figure 5 we consider a larger scale version of the example we described above. In this larger scale experiment, we generate 40 classes from 15 different functional groups. Each gene is randomly assigned to a gene expression subgroup. While in this case there are multiple marker sets that are suitable for discriminating among a given clustering, the probability of detecting a good marker set is negligible $(\mathrm{O}(1/(40)^{15}))$.

In this specific example we have 1000 cells and 8,798 gene ScGeneFit finds the number of markers of this example in 10-12 minutes in a standard 2018 MacBook Pro (or a Google Colab on a standard free account). In order to make our algorithm work in this larger scale setting we modify our method as explained in the next Section as well as in the methods Section of our manuscript.

In order to deal with thousands of genes we consider a different way to choose the set of constraints. Basically in this version we consider one variable per gene and constraints of the form $\|c_t - c_s\| > \Delta$ where c_t it the empirical center of the t-th class. We aim to have a number of constraints of the order of the number of classes, therefore we don't consider the constraints over all pairs of centers but $O(1)$

Functional group	subgroup	G_1	G_2	G_3	G_4	G_5
A	A_1	0	0	-	-	-
	A_2	0	1	-	-	-
	A_3	1	0	-	-	-
	A_4	1	1	-	-	-
B	B_1	-	-	2	-	-
	B_2	-	-	3	-	-
C	C_1	-	-	-	0	0
	C_2	-	-	-	2	0
	C_3	-	-	-	0	2
	C_4	-	-	-	2	2

Supplementary Table 1: In this example we consider 3 functional groups, A, B, C. Across all groups we consider a homoskedastic variance σI_k with $\sigma = 0.5$ where I_2 is the identity matrix and k is the number of genes determining the group. Group A is determined by two genes with multivariate normal gene expression with means [0,0], [0,1], [1,0], and [1,1], group B is determined by one gene with multivariate normal gene expression means [2] (mode B_1) and [3] (mode B_2). Similarly, group C is determined by two genes with multivariate normal gene expression means [0,0], [2,0], [0,2], and [2,2]. The cell types are identified through functional group tuples (A_i, B_j, C_k) . In this example we have $32 = 4 * 4 * 2$ possible cell types. For instance, if we consider the cell types $T_1 = (A_1, B_1, C_4)$ and $T_2 = (A_2, B_2, C_3)$ it would suffice to use G_3 as a marker to distinguish both cell types. However, if we consider all possible 32 cell types one needs all 5 genes to distinguish them. We use this functional groups example in Supplementary Figures 2 and 3

constraints per center. This implementation is quite efficient but it relies on the underlying assumption that the classes are linearly separable, which is a reasonable assumption in high dimensions but may not be in lower dimensions. All these different implementations are documented in our released code.

In Supplementary Figure 5 we show the performance of scGeneFit in a larger scale problem and its comparison with one-vs-all.

CBMC and Zeisel datasets

In this Section we report the performance of the scGeneFit 'pairwise' and 'centers' methods on CBMC and Zeisel datasets in comparison with one-vs-all (Supplementary Tables 2 and 3, and Supplementary Figure 7). More results are presented in the main manuscript as well as the Github repository `https://github.com/solevillar/scGeneFit-python/`.

In Supplementary Table 2 K-nearest neighbor based metrics were used to evaluate the performance of the flat clustering. Due to the small number of points per subcluster this method is not suitable for evaluating the lower level of the hierarchy.

Precision Recall Values For Hierarchical Clustering

scGeneFit provides a method for marker selection according to a hierarchical clustering structure, described in the Methods section of the main manuscript. In Supplementary Figure 8 and Supplementary Table 4 we report its performance on Zeisel using hierarchical labels.

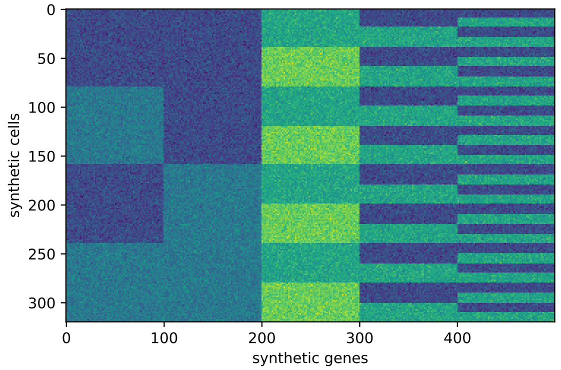

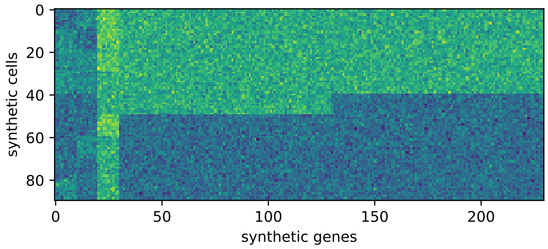

Supplementary Figure 2: We consider the functional groups model defined in Supplementary Table 1. In this model, a cell type is defined by its group (A_i, B_j, C_k) where $i = 1, 2, 3, 4$, $j = 1, 2$ and $k = 1, 2, 3, 4$. The gene expression G_s for $s = 1, 2, 3, 4, 5$ is drawn from a Gaussian distribution where the mean is determined by the type according to Table 1. (Left) we draw 10 cells from each of the 32 possible cell types. A random copy of each of the genes is drawn 100 consecutive times. In this example 5 markers are necessary and sufficient to distinguish all 32 classes. Note that even though all genes could technically be markers, the probability of selecting one from each class by selecting 5 genes at random is negligible. (Right) in this example we consider 9 cell types. The first 3 genes are repeated 10 times whereas the last two genes are repeated 100 times. The results of one-vs-all and scGeneFit for this synthetic data is shown in Supplementary Figure 3.

Detected Markers

scGeneFit identifies both cell-type specific markers (e.g., Pde1a, Cnr1, Enpp2, Mef2c), and markers that show broad patterns of behavior (e.g. the low expression of Tmsb4x is particular to astrocytes). Further interpreting the grammar governing this behavior is subject of future work, and is outside the scope of the current paper.

Supplementary References

[1] D. I. Inouye, E. Yang, G. I. Allen, and P. Ravikumar. A review of multivariate distributions for count data derived from the poisson distribution. *Wiley Interdisciplinary Reviews: Computational Statistics*, 9(3):e1398, 2017.

[2] E. Pierson and C. Yau. Zifa: Dimensionality reduction for zero-inflated single-cell gene expression analysis. *Genome biology*, 16(1):1–10, 2015.

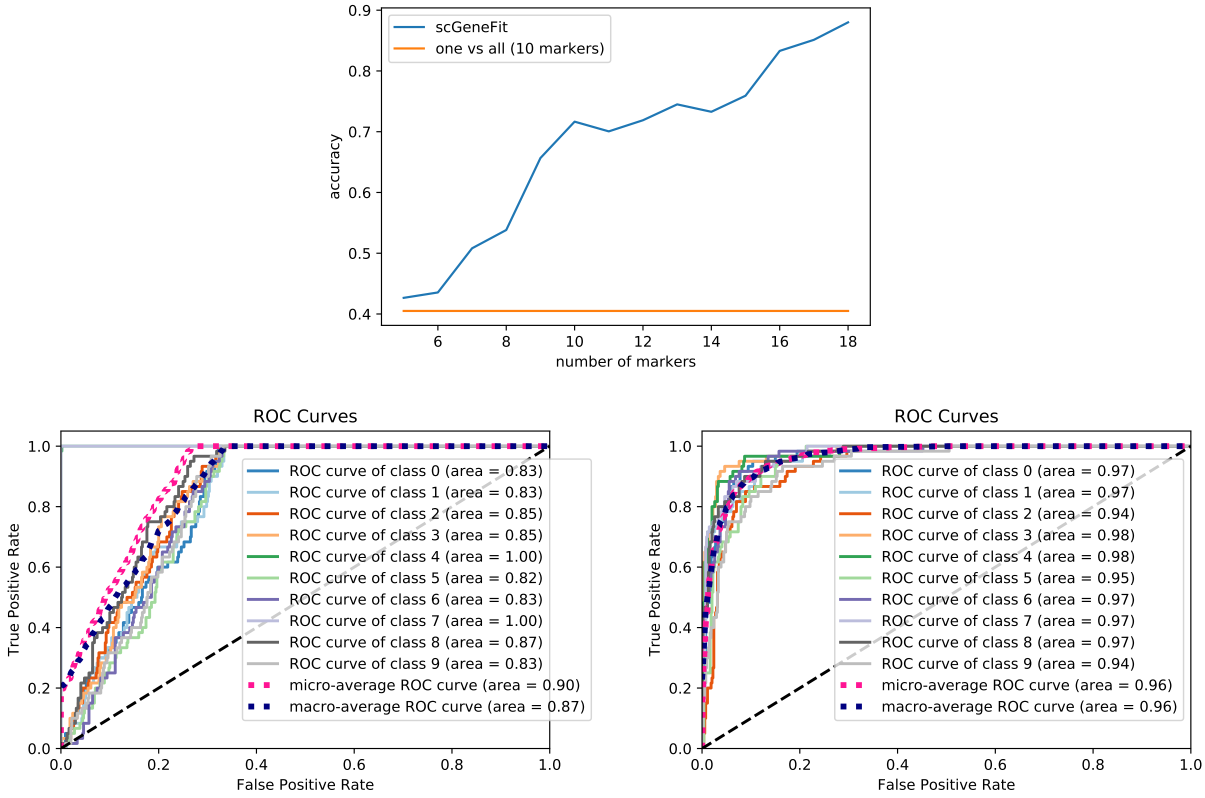

Supplementary Figure 3: **Small functional group simulation: Multivariate Normal Case.** We consider a toy dataset of 90 cells and 230 genes from 9 different cell types. The genes belong to one of the 5 different gene types described in Supplementary Figure 2 (Right) and Supplementary Table 1. **(Top left)** tSNE plot of the original data from Supplementary Figure 2 followed by tSNE plot of the data restricted to the markers selected by the one-vs-all procedure. We observe that some classes are well distinguished (for instance 3,4 and 5) but other classes are not for instance (0,1,2). This is made quantitative in the second row plot. **(Top right)**: a tSNE plot of the original data followed by tSNE plot of the data restricted to 9 markers selected by scGeneFit procedure. **(Second row)**: scGeneFit performance improves significantly as the number of markers is allowed to increase: we plot the classification accuracy of a K-nearest neighbors classifiers as a function of the number of markers **(Third row)**: Receiver operating characteristic curves for (left) one vs all, and (right) scGeneFit .

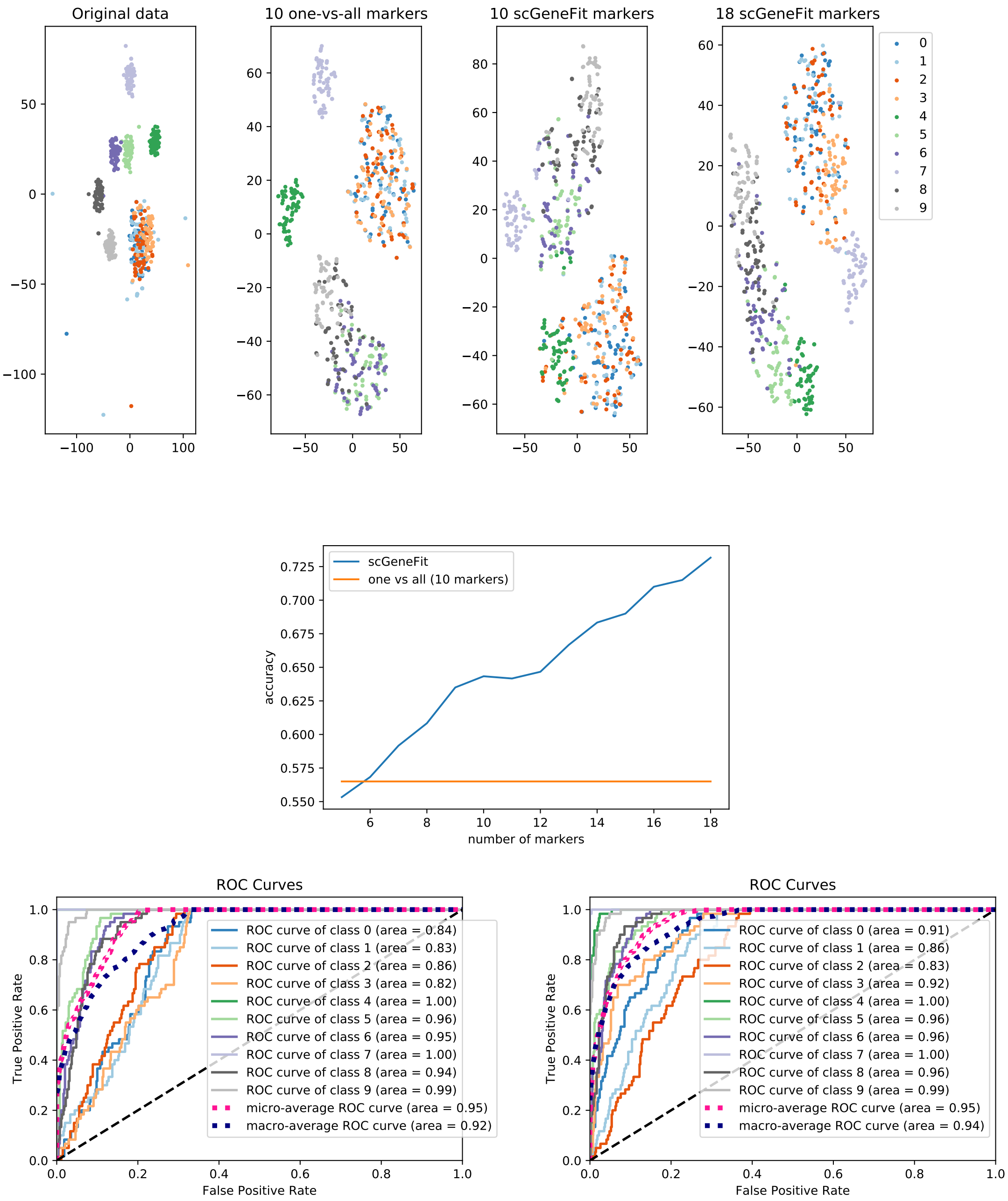

Supplementary Figure 4: **Small functional group simulation: Multivariate Poisson-Normal Case.** We consider a toy dataset of 90 cells and 230 genes from 9 different cell types. This model extends the multivariate normal functional group toy model in Supplementary Figure 2 to include a Poisson link. The Poisson link has a strong effect on the performance of the one-vs-all analysis. In contrast scGeneFit is robust to this transformation. **(Top left)**: a tSNE plot of the original data from Supplementary Figure 2 followed by tSNE plot of the data restricted to the markers selected by the one-vs-all procedure. We observe that some classes are well distinguished (for instance 3,4 and 5) but other classes are not for instance (0,1,2). This is made quantitative in the second row plot. **(Top right)**: a tSNE plot of the original data followed by tSNE plot of the data restricted to 9 markers selected by scGeneFit procedure. **(Second row)**: scGeneFit performance improves significantly as the number of markers is allowed to increase: we plot the classification accuracy of a K-nearest neighbors classifiers as a function of the number of markers **(Third row)** Receiver operating characteristic curves for (left) one vs all, and (right) scGeneFit .

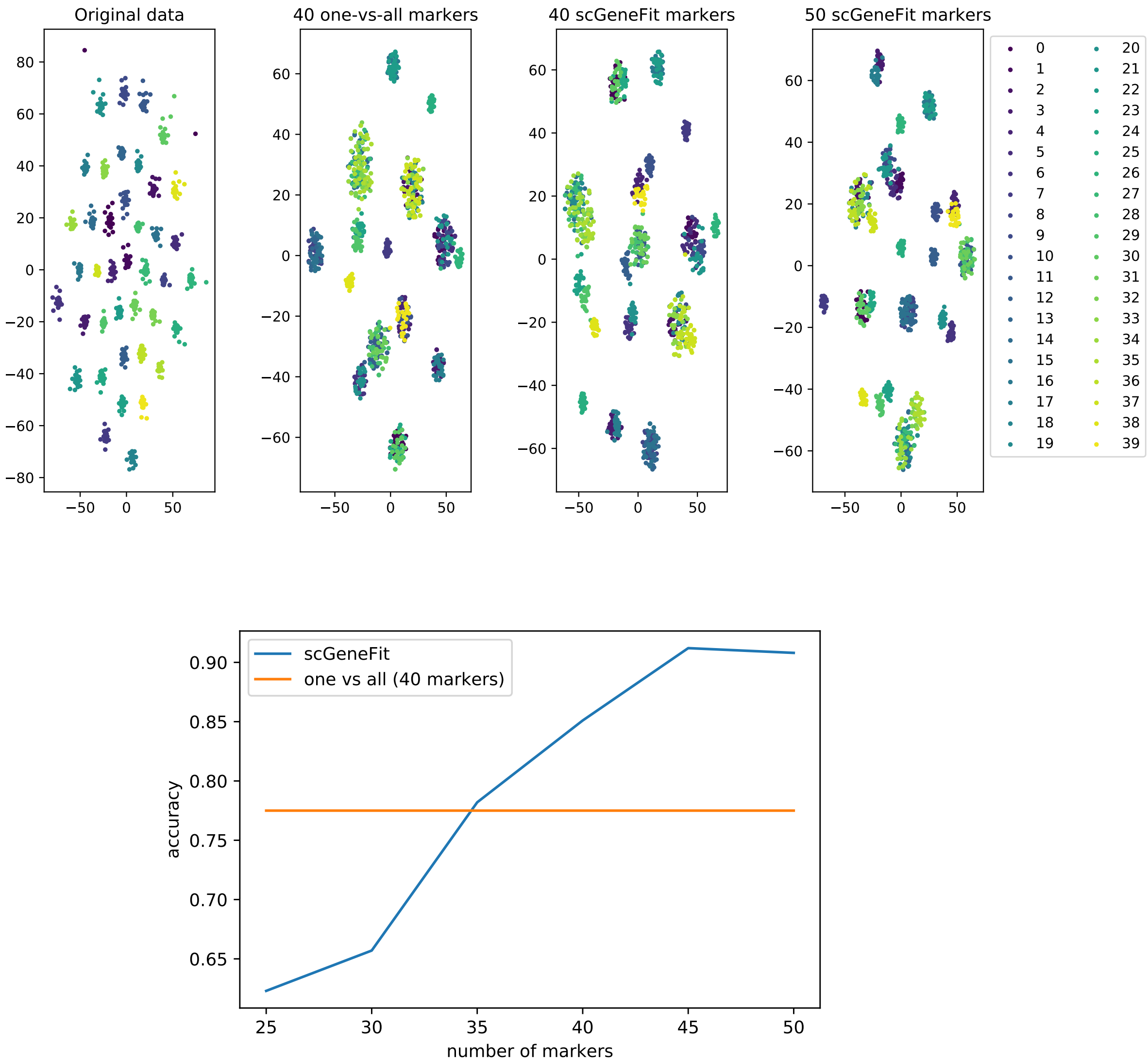

Supplementary Figure 5: **Scaled-up functional group experiment: Multivariate Normal Case.** We generated 40 classes from 15 different functional groups. We set each gene type to be repeated a random number of times between 10 and 1000. In this specific random example we have 1000 cells and 10,000 genes. Each run of the scGeneFit takes around 10 minutes in a Google Colab (standard account) (Top) Representation of the cells and genes as a matrix. (Middle) tSNE plots of (from left to right) original data, data restricted to the 40 markers selected by one vs all, data restricted to the 40 markers selected by scGeneFit, data restricted to 45 markers selected by scGeneFit. (Bottom) We report the classification accuracy of scGeneFit and one vs all.

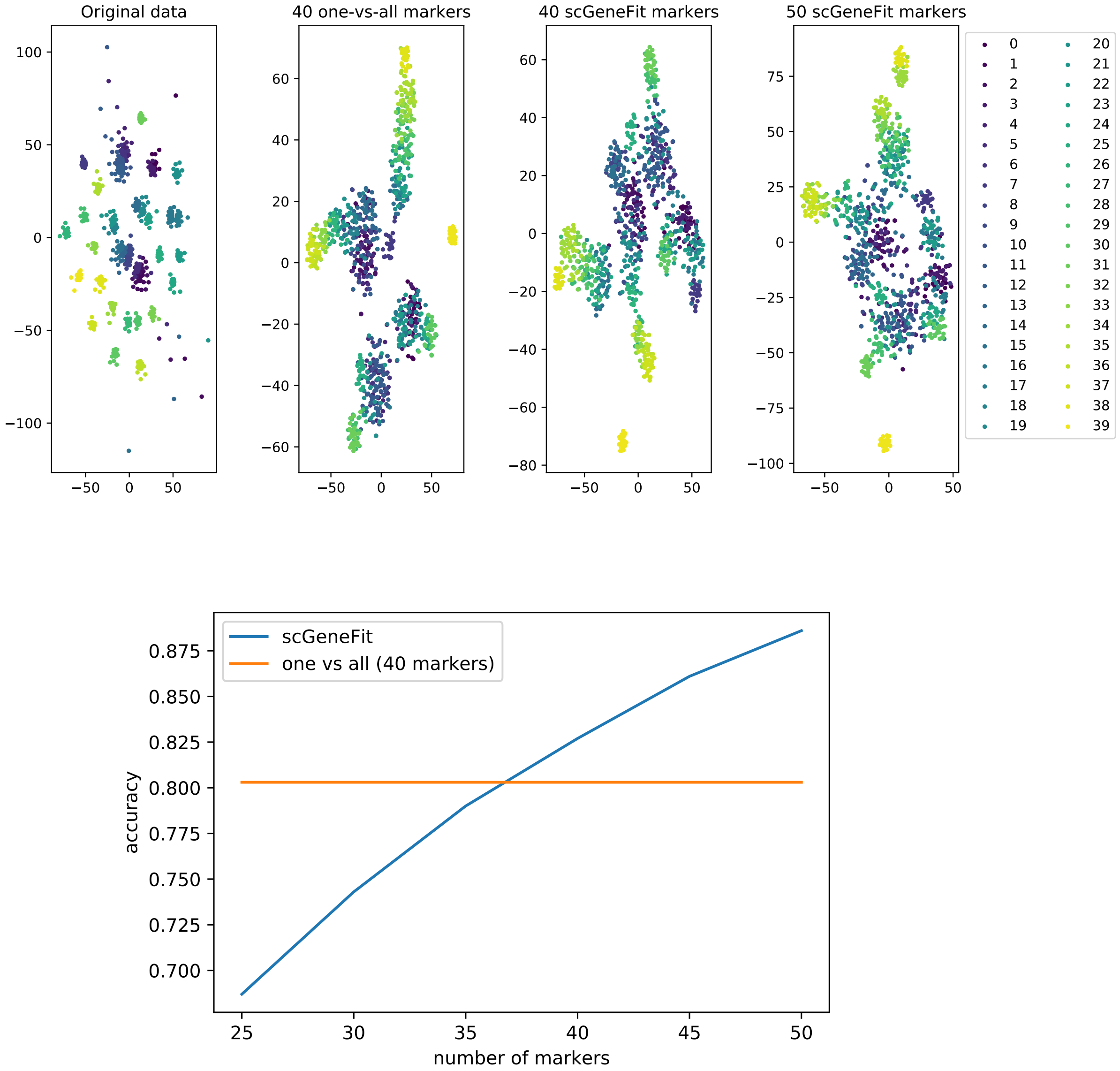

Supplementary Figure 6: **Scaled-up functional group experiment: Multivariate Poisson-Normal Case.** We generated 40 classes from 15 different functional groups. We set each gene type to be repeated a random number of times between 10 and 1000. In this specific random example we have 1000 cells and 10,000 genes. Each run of the scGeneFit takes around 10 minutes in a Google Colab (standard account) (Top) tSNE plots of (from left to right) original data, data restricted to the 40 markers selected by one vs all, data restricted to the 40 markers selected by scGeneFit, data restricted to 50 markers selected by scGeneFit. (Bottom) We report the classification accuracy of scGeneFit and one vs all.

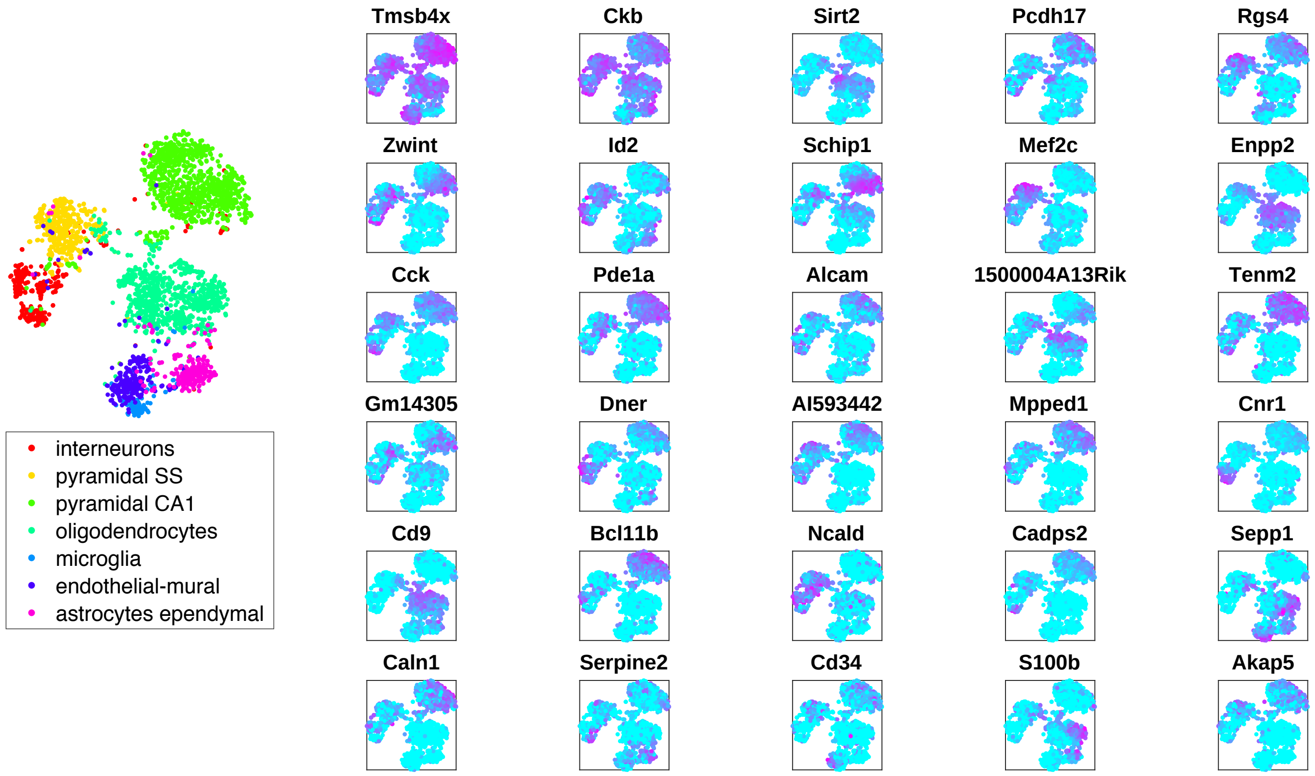

Supplementary Figure 7: Reference t-SNE plot of the main cell types in the Zeisel data set: interneurons, pyramidal SS cells, pyramidal CA1 cells, oligodendrocytes, microglia, endothelial-mural cells, and astrocytes (left). The 30 markers uncovered by scGeneFit 'pairwise' in the hierarchical setting. The markers are overlayed based on their expression – low (blue) to high (pink) – on the reference Zeisel t-SNE plot (right).

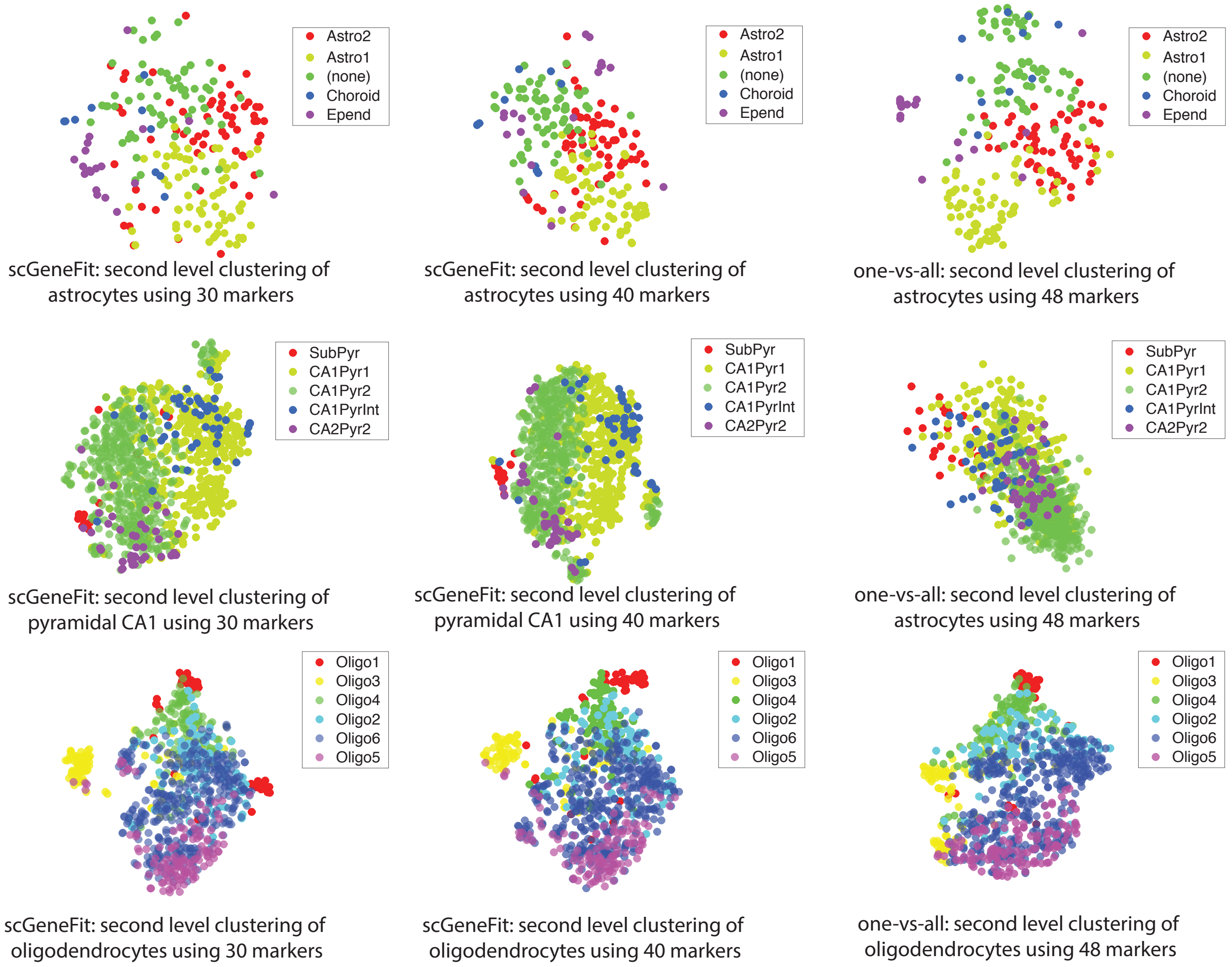

Supplementary Figure 8: Level two subclustering across three major groups: astrocytes, pyramidal CA1, and oligodendrocytes. t-SNE plots illustrate the performance achieved with scGeneFit with 30 and 40 is comparable with an one-vs-all approach using 48 markers.

Marker selection for CBMC

	**original data**	**random markers**		**one-vs-all**	**scGeneFit**		
markers	500	13	20	13	13	20	30
$K = 3$	7.47	19.77	14.08	9.90	9.40	8.47	8.05
$K = 5$	7.20	21.35	20.41	9.01	8.82	7.97	7.93
$K = 15$	7.85	23.48	17.68	9.01	8.59	8.28	7.93
k-means	14.99	22.45	15.55	11.60	10.92	10.70	10.84

Marker selection for Zeisel (first level cluster)

	**original data**	**random markers**		**one-vs-all**	**scGeneFit**		
markers	4000	7	40	7	7	30	40
$K = 3$	2.77	64.70	28.41	12.43	16.87	10.21	6.77
$K = 5$	3.44	57.82	25.75	11.87	15.65	8.54	5.88
$K = 15$	4.10	54.16	25.31	10.43	14.54	8.32	6.55
k-means	19.78	27.28	22.17	9.74	10.86	8.75	6.44

Supplementary Table 2: Percentage of misclassified CBMC cells (top table) and Zeisel cells (bottom table) from K-nearest neighbor and k-means after compressing with random markers, markers chosen as most significantly associated with a cluster and scGeneFit with 'pairwise' method (a lower score is a better score). (The Id column takes identity to be the "compression" operator.) For the first rows we split the data in 70% training (6032 cells for CBMC, 2104 for Zeisel) and 30% test (2585 cells for CBMC, 901 for Zeisel). We train a K-nearest neighbor classifier on the compressed training set, and report its misclassification error in the compressed test set. The last row considers the smallest misclassification error of k-means clustering among 10 runs of k-means with different random seeds. Due to the high variance in the number of points per cluster, k-means is not a suitable clustering method for this CBMC.

scGeneFit: Precision Recall for CBMC

**cell type**	**precision**	**recall**	**f1 - score**	**support**
B	**0.97**	0.95	**0.96**	65
CD14+ Mono	0.97	0.74	0.84	639
CD16+ Mono	0.63	**0.94**	**0.75**	63
CD34+	**0.94**	0.97	**0.96**	35
CD4 T	**0.95**	**0.76**	**0.85**	752
CD8 T	**0.15**	**0.60**	**0.25**	60
DC	**0.54**	1.00	**0.70**	21
Eryth	0.95	**0.90**	**0.93**	21
Mk	**0.40**	**0.28**	**0.33**	29
Mouse	1.00	0.96	0.98	168
NK	0.98	**0.92**	0.95	243
Unknown	0.23	**0.71**	0.35	51
pDC	0.89	1.00	0.94	8

one-vs-all: Precision Recall for CBMC

**cell type**	**precision**	**recall**	**f1 - score**	**support**
B	0.95	0.95	0.95	65
CD14+ Mono	0.97	**0.75**	**0.85**	639
CD16+ Mono	**0.67**	0.79	0.72	63
CD34+	0.88	**1.00**	0.93	35
CD4 T	0.91	0.64	0.75	752
CD8 T	0.11	0.55	0.18	60
DC	0.32	1.00	0.48	21
Eryth	0.95	0.86	0.90	21
Mk	0.28	0.24	0.26	29
Mouse	1.00	**0.98**	**0.99**	168
NK	**0.99**	0.91	0.95	243
Unknown	**0.24**	0.65	0.35	51
pDC	**1.00**	1.00	**1.00**	8

Supplementary Table 3: Metrics for evaluating the performance of scGeneFit on CBMC. We find the markers using scGeneFit and one-vs-all on a training set of 75% of the dataset (selected at random), and we evaluate its performance on the remaining 25%. The best performers are shown **bolded**.

scGeneFit: Precision Recall for supervised hierarchical clustering information (first level cluster)

**cell type**	**precision**	**recall**	**f1 - score**	**support**
cell type	precision	recall	f1-score	support
astrocytes	**0.81**	0.81	**0.81**	53
endothelial	0.66	**0.88**	**0.76**	51
interneurons	**0.85**	0.77	**0.81**	66
microglia	**0.71**	0.68	**0.70**	22
oligodendrocytes	**0.96**	0.94	**0.95**	189
pyramidal CA1	0.95	**0.91**	**0.93**	253
pyramidal S1	**0.90**	**0.94**	**0.92**	118

one-vs-all: Precision Recall for supervised hierarchical clustering information (first level cluster)

**cell type**	**precision**	**recall**	**f1 - score**	**support**
astrocytes	0.65	**0.85**	0.74	53
endothelial	**0.71**	0.47	0.56	51
interneurons	0.70	**0.80**	0.75	66
microglia	0.44	0.68	0.54	22
oligodendrocytes	0.89	**0.99**	0.94	189
pyramidal CA1	0.96	0.88	0.92	253
pyramidal S1	0.88	0.71	0.79	118

Supplementary Table 4: Metrics for evaluating the performance of scGeneFit on a hierarchical dataset. We find the markers using scGeneFit and one-vs-all on a training set of 75% of the dataset (selected at random), and we evaluate its performance on the remaining 25%. The metrics (precision, recall, f1 score) are computed at the first level of the hierarchy. scGeneFit improves on most categories. The best performers are shown **bolded**. It is worth mentioning that the cluster labels of the Zeisel dataset were obtained with a one-vs-all analysis in mind.
